# The Influence of Climate and Livestock Reservoirs on Human Cases of Giardiasis

**DOI:** 10.1007/s10393-018-1385-7

**Published:** 2018-10-22

**Authors:** Ariel Brunn, David N. Fisman, Jan M. Sargeant, Amy L. Greer

**Affiliations:** 1 Department of Population Medicine, Ontario Veterinary College, University of Guelph, 50 Stone Road East, Guelph, ON N1G 2W1 Canada; 2 Department of Epidemiology, Dalla Lana School of Public Health, University of Toronto, Toronto, ON Canada; 3 Centre for Public Health and Zoonoses, University of Guelph, Guelph, ON Canada; 4 Arrell Food Institute, University of Guelph, Guelph, ON Canada

**Keywords:** *Giardia*, Giardiasis, Climate, Watershed, One health, Case crossover, Poisson

## Abstract

*Giardia duodenalis* is an intestinal parasite which causes diarrhoeal illness in people. Zoonotic subtypes found in livestock may contribute to human disease occurrence through runoff of manure into multi-use surface water. This study investigated temporal associations among selected environmental variables and *G. duodenalis* occurrence in livestock reservoirs on human giardiasis incidence using data collected in the Waterloo Health Region, Ontario, Canada. The study objectives were to: (1) evaluate associations between human cases and environmental variables between 1 June 2006 and 31 December 2013, and (2) evaluate associations between human cases, environmental variables and livestock reservoirs using a subset of this time series, with both analyses controlling for seasonal and long-term trends. Human disease incidence exhibited a seasonal trend but no annual trend. A Poisson multivariable regression model identified an inverse association with water level lagged by 1 month (IRR = 0.10, 95% CI 0.01, 0.85, *P* < 0.05). Case crossover analysis found varying associations between lagged variables including livestock reservoirs (1 week), mean air temperature (3 weeks), river water level (1 week) and flow rate (1 week), and precipitation (4 weeks). This study contributes to our understanding of epidemiologic relationships influencing human giardiasis cases in Ontario, Canada.

**Electronic supplementary material:**

The online version of this article (10.1007/s10393-018-1385-7) contains supplementary material, which is available to authorized users.

## Introduction

Enteric illness caused by the intestinal parasite *Giardia duodenalis* (*Gd*) is reportable in Canada. The parasite is the third-leading cause of enteric disease in the province of Ontario with an average provincial incidence of 9.9 cases per 100 000 person-years reported between 2011 and 2015 (Public Health Ontario 2017). Many patients remain asymptomatic or are only transiently afflicted causing underreporting of the true prevalence. Symptomatic patients contribute to the estimated 89.5 million disability-adjusted-life-years lost world-wide due to diarrheal disease (Conlan and Lal 2015; Murray et al. 2012). However, correlations between socioeconomic factors such as lack of plumbing and an increased risk of infection may explain why giardiasis has a higher prevalence in lower-income countries (Silva et al. 2009). Symptomatic cases of giardiasis may exhibit mild abdominal discomfort, diarrhoea, vomiting and weight loss, however, most infections are self-resolving.

*Gd* is composed of eight assemblages (A–H), which are genetic groupings with varying degrees of host-specific pathogenicity. Assemblages A and B primarily cause disease in humans (Yaoyu and Xiao 2011) but have zoonotic potential as they have been isolated from cattle, swine, dogs and cats, as well as some wild mammals (Cacciò and Ryan 2008; Farzan et al. 2011; Yaoyu and Xiao 2011). Assemblage types C–H have limited host ranges of domestic and wild animals, but rare occurrences in people have been reported (Yaoyu and Xiao 2011). Zoonotic assemblages in livestock contribute to human disease through direct exposure or through manure contaminated water sources used for recreation or drinking water (Hunter and Thompson 2005; Farzan et al. 2011; Abdel-Moein and Saeed 2016). Ontario dairy cattle and pigs have been shown to harbour zoonotic subtypes of *Gd* (Coklin et al. 2007; Dixon et al. 2011; Farzan et al. 2011) and cysts shed in host faeces are immediately infective in the environment. The low specific gravity of cysts along with high precipitation enhances transport from soil into water sources (Erickson and Ortega 2006; Wilkes et al. 2011). Cysts are well adapted for wet and humid conditions, remaining infective in water with a temperature of 0–4 °C for 84 days (Yaoyu and Xiao 2011). Warmer air temperatures lead to increased recreational use of water sources by people and hydrological characteristics of watersheds such as water flow and level can affect the suspension and sedimentation rate of cysts in surface waters (Erickson and Ortega 2006) facilitating *Giardia* cyst ingestion by people (Thomas et al. 2006; Peng et al. 2008). The most common risk factors for human infection in Ontario include ingestion of contaminated water (e.g. drinking or recreational water), direct transmission via the faecal-oral route from infected people or animals, or consumption of contaminated food such as unwashed vegetables (Public Health Ontario 2017). Although outbreaks of giardiasis have been reported globally, they are uncommon in Ontario with a recent descriptive study suggesting that 99% of G*iardia* cases are not linked epidemiologically (Keegan et al. 2009).

The seasonal pattern of human *Giardia* incidence has been reported with annual peaks in the late summer and early autumn (Addiss et al. 1992; Greig et al. 2001; Naumova et al. 2007; Keegan et al. 2009; Lal et al. 2012). This seasonal fluctuation, combined with the ability of *Giardia* to persist in the environment, is highly suggestive of a transmission cycle that is sensitive to climate and hydrological factors (Fisman 2007; Greer et al. 2008; Ng et al. 2008). Therefore, our a priori hypothesis is that environmental and hydrological conditions are associated with the occurrence of sporadic human *Giardia* cases within southern Ontario. Understanding associations between environment, livestock reservoirs, and human disease may enhance prevention and control strategies. In this respect, the use of a one health approach is appropriate for the study of *Giardia* to avoid simplifying complex relationships (Bouley et al. 2014). The one health concept is a framework that recognizes the interconnectedness between the health of humans, animals and the environment (Bouley et al. 2014). The objectives of this study were to identify temporal associations between environmental conditions, livestock *Giardia* reservoirs and human giardiasis incidence using an integrated time series (2006–2013) from the Waterloo Health Region (WHR) in Ontario, Canada, and to compare the results obtained using two different methods that account for confounding due to seasonality and annual trends.

## Methods

### Sources of Data

#### Human Case Data

**Figure 1:**
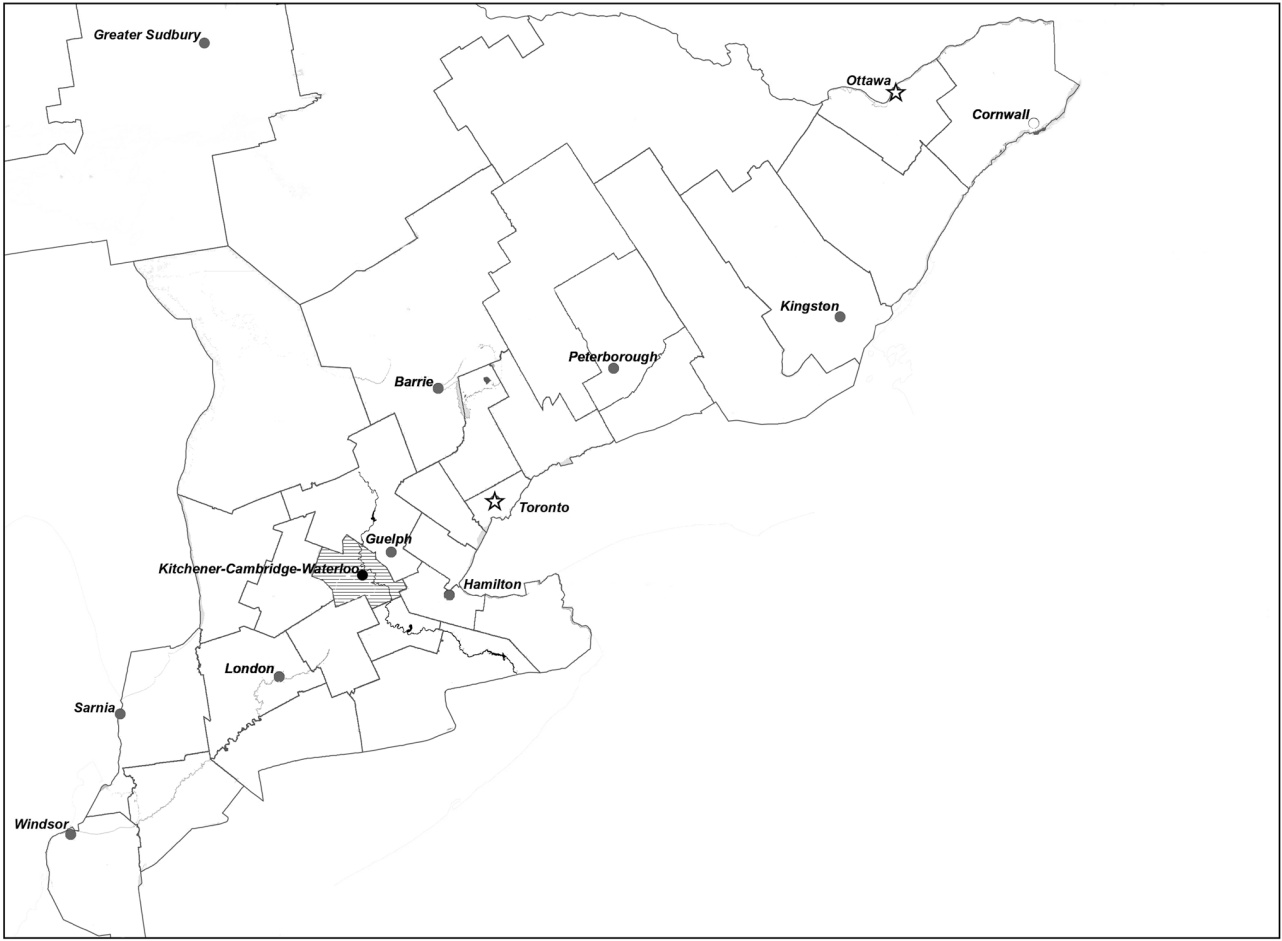
Map of the Waterloo Health Region, Ontario, Canada (WHR—shaded region) where the observational data were collected. The Grand River can be seen as a river that flows from north to south through the health region. * = Location of Waterloo International Airport where Environment Canada weather conditions (mean temperature, total precipitation) were collected; ǂ = location of Doon, Waterloo County, where Environment and Climate Change Canada hydrology conditions were collected on the Grand River, ON, CA.

The Canadian province of Ontario is made up of 36 public health units and all confirmed cases of *Gd* are reported to the provincial Integrated Public Health Information System (iPHIS). The WHR encompasses part of the multi-use Grand River watershed located in southwestern Ontario (Fig. 1). In 2011, the 1369 km^2^ region was densely populated with 507 096 people, 815 cattle farms and 210 swine farms (Statistics Canada 2011).

Confirmed cases of *Gd* are reported to iPHIS based on the finding of cysts or trophozoites in stool samples from patients with compatible clinical signs (after 2009, demonstration of *Gd* antigen by enzyme immunoassay or immunochromatographic test was also accepted in place of microscopy) (Ontario Ministry of Health and Long-Term Care 2016). The case report date could be the date of symptom onset, the specimen submission date, the lab report date or the date the case was reported to the database. Underreporting of enteric diseases is considerable because asymptomatic, transient or mildly affected patients may not attend a health care provider (Murphy et al. 2016). Therefore, every unique submitted case to iPHIS was used to maximize the study population. This study population is thought to be representative of the patients suffering impairment from disease. Cases who reported travel outside of the province of Ontario during the disease incubation period, or who could not confirm their travel history during the incubation period, were excluded. All cases in the dataset for the time series were identified as sporadic based on the provincial outbreak numbering system. Cases not identified by the local public health unit as being linked to an outbreak were considered to have occurred independently of other cases and coded with a unique identifier.

#### Climate and Hydrology Data

The WHR has a temperate climate consisting of distinct seasons made up of warm summers and cold winters with below freezing temperatures (Peel et al. 2007). Weather and hydrology observations for this region were measured daily over the study period from 1 June 2006 to 31 December 2013. The environmental variables included mean air temperature (°C) and total precipitation (mm), river water level (m) and river water flow rate (m^3^/s) (Environment and Climate Change Canada 2016a, b). Studies on the physical characteristics of *Giardia* have found that temperature is an important parameter influencing survival and recovery of viable cysts (Erickson and Ortega 2006). The focus of this study on waterborne transmission meant that precipitation, water flow and water level were variables of interest based on our a priori hypothesis. Weather was recorded at the Region of Waterloo International Airport, Waterloo, Ontario; daily water flow and water level observations were collected on the Grand River at Doon, Waterloo County, Ontario (Environment and Climate Change Canada 2016a, b).

#### Livestock Reservoir Data

The pilot site of the FoodNet sentinel surveillance program of the Public Health Agency of Canada operated in the WHR between 2005 and 2014. Farm level surveillance of livestock manure samples was conducted. Each month, one or more farm type (dairy, beef or swine farm) was visited and 3 fresh, pooled manure samples and one pooled manure pit sample were collected (Public Health Agency of Canada 2010). Each fresh sample comprised manure from five animals, representing different ages, and the manure pit sample comprised up to five subsamples collected from different depths. Samples were tested by microscopy and PCR. At least one positive sample per farm per collection date underwent further molecular subtyping to determine the assemblage (Public Health Agency of Canada 2010). PCR and/or microscopy results for manure samples were recorded as positive, negative, or not done. A farm was labelled positive if at least one sample from the farm on a given collection date was positive for *Gd* by PCR and/or microscopy. Numeric cyst counts were not performed. Aggregated counts of *Giardia*-positive farms per manure testing date were compiled to form a time series of positive livestock reservoirs in the region between 1 June 2006 and 31 December 2008.

### Data Organization

The study was separated into two parts to reflect the two objectives: Objective I evaluated associations between human cases and environmental variables between 1 June 2006 and 31 December 2013. For objective II, a subset of observations from Objective I collected between 1 June 2006 and 31 December 2008 were merged with the time series of *Giardia*-positive farms (livestock reservoir data) to create a dataset that integrated human, animal and environmental data.

### Statistical Analysis

#### Seasonality

Seasonal fluctuation of human *Giardia* incidence was assessed for statistical significance by linear combination of sine and cosine terms. We employed methods previously described by Ng et al. (2008) to use monthly sine and cosine variables to assess seasonality and a year term to evaluate annual trends:

$$ E\left( Y \right) = \exp \left( {\alpha + \beta 1\left( {\text{year}} \right) + \beta 2\left( {\sin \left( {2\pi {\text{ month}}/12} \right)} \right) + \beta 3\left( {\cos \left( {2\pi {\text{ month}}/12} \right)} \right)} \right) $$

where *α* is a constant, *β* is a regression coefficient for year or month and *E*(*Y*) is the expected case count for a given month (Ng et al. 2008).

Annual variation and statistical significance of the seasonal smoothing terms were evaluated using a Poisson regression model with no predictor variables included. Significant smoothing terms (*P* ≤ 0.05) were included in univariable analysis models of predictor variables; oscillating seasonal smoothers (sine and cosine terms) and the year variable were forced into all multivariable models to adjust for the expected confounding effects of seasonal and annual trends.

#### Multivariable Regression Models

For each objective, Poisson regression analysis was used to examine temporal associations among average monthly weather conditions (temperature and precipitation), hydrological conditions (river flow and level), and monthly aggregated *Giardia*-positive farms (Objective II) on monthly aggregated human case counts. Canadian census information provided the total annual population at risk to determine the incidence rate ratio of giardiasis in people in WHR. A 1-month lag period for environmental and livestock reservoir variables was chosen as it included the incubation period of *Gd* in humans (approximately 7–14 days) as well as time for the pathogen to move from livestock and the environment into the human population. Correlation between predictor variables was assessed (cutpoint > 0.8) and highly correlated pairs of variables reduced by eliminating one of the variables from further analysis. After univariable analysis (adjusted for statistically significant seasonal smoothers), monthly averaged environmental variables and monthly aggregated *Giardia*-positive farms that had incidence rate ratios (IRRs) that met a liberal *P* value (*P* ≤ 0.2) were brought forward into a multivariable Poisson regression model and a backward selection process was used to determine the final multivariable model (*P* ≤ 0.05). Scatter plots of predicted outcomes and Anscombe residuals were created to assess outliers and covariate patterns with high influence on the model.

#### Case Crossover Models

A case crossover design was used to assess the impact of acute environmental and livestock exposures on daily human case counts. This approach is useful for rare diseases with a short incubation period and intermittent exposures that create different risk periods through which subjects pass temporally (Maclure 1991; Levy et al. 2001; Ng et al. 2008). The self-matching, case crossover design compares the exposure status immediately before a case occurs to the self-matched exposure status during a control period which is randomly selected to occur prior to, after, or spanning the hazard period (Levy et al. 2001). A time-stratified, 4:1 matched design was used to match four control periods by day of week to the case report date within each 4-week stratum. Conditional logistic regression models were used to assess statistical associations between same week and 1–4-week-lagged exposures and daily human case counts. Environmental variables were ranked from lowest to highest within each stratum to further elucidate which exposure most contributed to risk of human case development. A distributed lag model was used to account for transmission of the pathogen through the environment and the average incubation period in humans.

Case crossover associations were measured as odds ratios (OR). Statistically significant associations between human cases and exposures reported during the concurrent week (i.e. exposures with no lag period) were not considered to be biologically feasible, given the mean incubation period for *Gd* in people is 7–14 days. All statistical analysis was conducted using STATA 14.0 (STATA Corporation, College Station, TX).

## Results

### Objective I: Environmental Variables and Human Case Counts: 2006–2013

**Figure 2:**
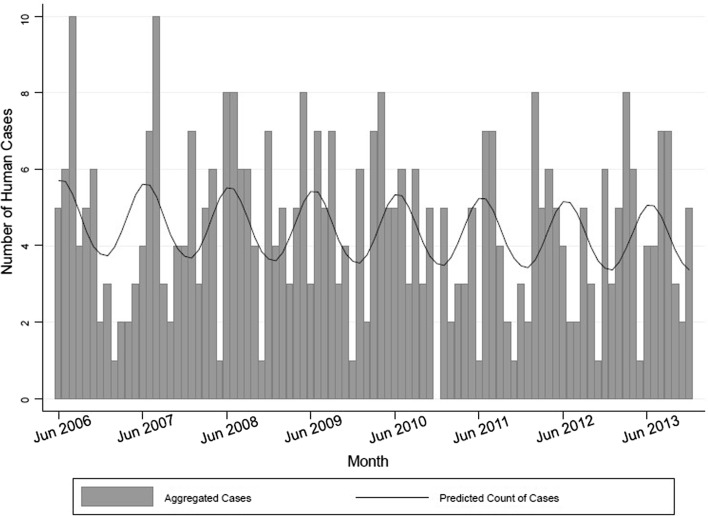
Predicted and observed human *Giardia duodenalis* cases from the Waterloo health region, Ontario, 2006–2013. Observed monthly human case counts are represented by the grey bars. Model predicted monthly human case counts are represented by the oscillating black line.

Between 1 June 2006 and December 31st there were 403 cases of giardiasis reported to iPHIS by the WHR. A reported symptom onset date was included for 304 (75%) of the cases. The average annual rate of reported cases was 10.8 cases per 100 000 person-years. Monthly human case counts showed a late summer and early autumn peak (Fig. 2). High correlation (> 0.8) between water level and water flow meant that only water level was examined in multivariable regression analysis.

A statistically significant seasonal pattern (Fig. 2) indicated increased human case occurrence coinciding with the late summer and early autumn period (*P* value for linear combination of sine and cosine seasonal estimators = 0.01). No annual trends were identified (*P* > 0.05).

#### Poisson Regression Analysis

Univariable regression analysis found that human disease incidence was inversely associated with water level of the previous month only (IRR = 0.43, 95% CI 0.18, 1.00, *P* = 0.05) (Table 1). When this environmental exposure was applied to the main effects model adjusted for seasonal predictors and year, it was not statistically significant (*P* = 0.06).

**Table 1:** Univariable Analysis^a^ and Case Crossover Results for Objective I: Environmental Exposures and Human Case Counts, 2006–2013

Exposure variable		Univariable regression model				Exposure variable			Case crossover analysis			
		IRR	*P* value (*P* ≤ 0.2)	95% CI					OR	*P* value (*P* ≤ 0.05)	95% CI	
*Climate*	*Lag period*					*Climate*	*Tertile*	*Lag period* ^c^				
Mean air temperature (°C)	No lag	1.01	0.344	0.99, 1.12		Mean air temperature (°C)	**Lowest**	**3** **weeks**	**0.75**	**0.014**	**0.60, 0.94**	
	1 month	1.00	0.645	0.99, 1.01			Medium	3 weeks	1.18	0.200	0.92, 1.51	
							Highest	3 weeks	1.22	0.118	0.95, 1.57	
Total precipitation (mm)	No lag	1.00	0.966	0.93, 1.08		Total precipitation (mm)	Lowest	–	–	–	–	–
	1 month	0.99	0.831	0.92, 1.07			Medium	–	–	–	–	–
							Highest	–	–	–	–	–
*Hydrology*						*Hydrology*						
Water flow (m^3^/s)^b^	No lag	–	–	–	–	Water flow (m^3^/s)	Lowest	1 week	0.95	0.680	0.76, 1.19	
	1 month	–	–	–	–		**Medium**	**1** **week**	**1.38**	**0.010**	**1.08, 1.76**	
							**Highest**	**1** **week**	**0.74**	**0.032**	**0.57, 0.97**	
Water level (m)	No lag	1.11	0.804	0.48, 2.60		Water level (m)	Lowest	1 week	0.91	0.417	0.73, 1.14	
	**1** **month**	**0.43**	**0.050**	**0.18, 1.00**			**Medium**	**1** **week**	**1.42**	**0.005**	**1.11, 1.82**	
							Highest	1 week	0.77	0.060	0.59, 1.01	

Bold values are statistically significant

^a^Univariable model adjusted for cosine seasonal oscillator term (*P* < 0.05).

^b^No results due to collinearity (cutpoint > 0.8) between water flow and water level.

^c^Results for variables with at least one statistically significant result shown; complete results available in Supplement 3.

#### Case Crossover Analysis

Case crossover analysis found that the water level and flow rate of the Grand River 1 week prior to human cases was associated with an increased risk of giardiasis in the WHR (Table 1). Within-stratum coolest mean air temperatures (lowest tertile) increased the odds of human case occurrence 3 weeks later (OR 0.75, 95% CI 0.60, 0.94, *P* = 0.014) (Table 1).

### Objective 2: Environmental Variables, Livestock Reservoirs and Human Case Counts, 2006–2008

Between 1 June 2006 to 31 December 2008, 145 human cases were reported to iPHIS for the WHR representing an average rate of 11.6 cases per 100 000 person-years. There were 107 (73%) recorded cases with a reported date of symptom onset. Between 1 June 2006 and 31 December 2008, 335 manure samples were collected from 86 unique cattle and swine farms. 222 (66%) manure samples tested positive by either microscopy or PCR, or both. Assemblage typing data was restricted to 128 positive samples and identified 30 (23%) samples as assemblages A or B, the remaining samples were typed as assemblage E.

Similar to the results of Objective I, the truncated dataset also exhibited a trend of seasonal periodicity for human cases (*P* value for linear combination of sine and cosine seasonal estimators = 0.002) but no year on year trend (*P* > 0.05).

#### Poisson Regression Analysis

For the truncated time series, univariable regression analysis (controlling for seasonality) found that the odds of becoming a case were associated with water level (IRR = 6.18, 95% CI 1.22, 31.45, *P* = 0.03), 1-month-lagged water level (IRR = 0.25, 95% CI 0.04, 1.65, *P* = 0.15), 1-month-lagged mean temperature (IRR = 0.93, 95% CI 0.84, 1.02, *P* = 0.12), and 1-month-lagged total precipitation (IRR = 1.10, 95% CI 0.99, 1.21, *P* = 0.07) (Table 2). A univariable model using the number of *Giardia*-positive farms as the predictor found that *Giardia*-positive farms were not associated with human cases (*P* > 0.05). The final multivariable model controlling for seasonality and year, found statistically significant associations with water level and 1-month-lagged water level (IRR = 9.80, 95% CI 1.69, 56.71, *P* = 0.01; IRR = 0.10, 95% CI 0.01, 0.85, *P* = 0.04) (Table 2).

**Table 2:** Univariable^a^ and Multivariable^b^ Regression Analysis and Case Crossover Results, Objective II: Environmental Exposures, Livestock Reservoirs and Human Case Counts, 2006–2008

Exposure variable		Univariable regression model				Exposure variable			Case crossover analysis				
		IRR	*P* value (*P* ≤ 0.2)	95% CI					OR	*P* value (*P* ≤ 0.05)	95% CI		
*Climate*	*Lag period*					*Climate*	*Tertile*	*Lag period* ^d^					
Mean air temperature (°C)	No lag	1.02	0.624	0.93, 1.13		Mean air temperature (°C)	**Lowest**	**3** **weeks**	**0.56**	**0.004**	**0.38, 0.83**		
	**1** **month**	**0.93**	**0.120**	**0.84, 1.02**			**Medium**	**3** **weeks**	**1.58**	**0.029**	**1.05, 2.38**		
							Highest	3 weeks	1.32	0.188	0.87, 2.00		
Total precipitation (mm)	No lag	1.02	0.670	0.92, 1.13		Total precipitation (mm)	Lowest	4 weeks	0.94	0.733	0.64, 1.37		
	**1** **month**	**1.10**	**0.070**	**0.99, 1.21**			Medium	4 weeks	1.49	0.060	0.98, 2.27		
							**Highest**	**4** **weeks**	**0.60**	**0.038**	**0.37, 0.97**		
*Hydrology*						*Hydrology*							
Water flow (m^3^/s)^c^	No lag	–	–	–	–	Water flow (m^3^/s)	Lowes	–	–	–	–	–	
	1 month	–	–	–	–		Medium	–	–	–	–	–	
							Highest	–	–	–	–	–	
Water level (m)	**No lag**	**6.18**	**0.030**	**1.22, 31.45**		Water level (m)	Lowest	–	–	–	–	–	
	**1** **month**	**0.25**	**0.150**	**0.04, 1.65**			Medium	–	–	–	–	–	
							Highest	–	–	–	–	–	
						Livestock reservoir	–	**1** **week**	**1.65**	**0.001**	**1.23, 2.22**		

Regression variable		Multivariable regression model		
		IRR	*P* value (*P* ≤ 0.05)	95% CI
*Lag period*				
**Cosine month**	**–**	**0.68**	**0.005**	**0.52, 0.89**
Sine month	–	0.73	0.070	0.51, 1.03
Year	–	1.04	0.743	0.82, 1.31
**Water level**	**–**	**9.80**	**0.010**	**1.69, 56.71**
**Water level**	**1** **month**	**0.10**	**0.035**	**0.01, 0.85**

Bold values are statistically significant

^a^Univariable model adjusted for sine and cosine seasonal oscillator terms (*P* = 0.01, *P* = 0.05).

^b^Multivariable model adjusted for sine and cosine seasonal oscillator terms and year variable (forced into the model).

^c^No results due to collinearity (cutpoint > 0.8) between water flow and water level.

^d^Results for variables with at least one statistically significant result shown; complete results available in Supplement 3.

#### Case Crossover Analysis

**Figure 3:**
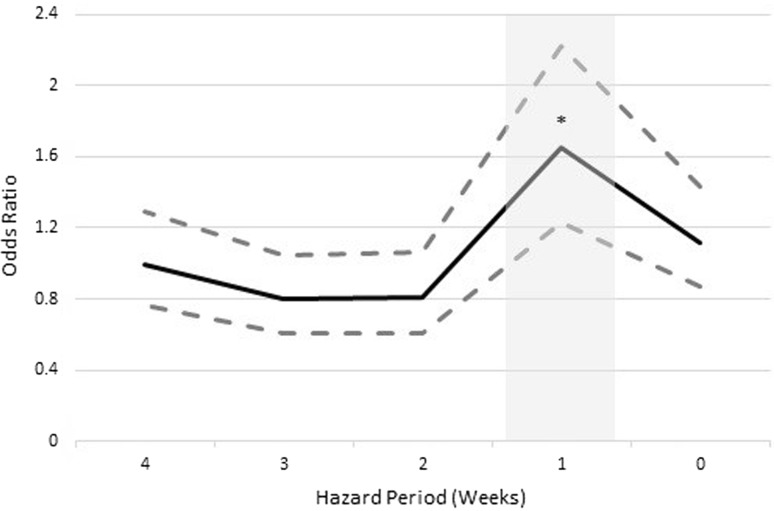
Case crossover results evaluating associations between the presence of weekly aggregated livestock reservoirs of *G. duodenalis* and human cases of *Giardia duodenalis* in the Waterloo health region, Ontario, 2006–2008. The hazard period is a 4-week period preceding a human case during which weekly aggregated exposures are assessed for influence on the odds of a human case occurring on day 0 (far right of the x-axis). Solid line = weekly odds ratio, dashed lines = upper and lower 95% confidence limits, * = statistically significant odds ratio (*P* ≤ 0.05), grey shaded box = time within the hazard period that precedes a human case with a statistically significant exposure.

Case crossover analysis of the truncated time series that included data on livestock reservoirs found associations between mean temperature and increased odds of human cases occurring 3 weeks later (Table 2), whereas decreases in temperature (lowest tertile) for the same lag period reduced the risk of infection (OR 1.58, 95% CI 1.05, 2.38, *P* = 0.029; OR 0.56, 95% CI 0.38, 0.83, *P* = 0.004). Livestock reservoirs lagged by one week increased human giardiasis risk (OR 1.65, 95% CI 1.23, 2.22, *P* = 0.001) (Fig. 3). Increasing levels of precipitation decreased the odds of an individual becoming a case 4 weeks later (OR 0.6, 95% CI 0.37, 0.97, *P* = 0.038) (Table 2).

## Discussion

This study contributes to our understanding of the relationship between environmental factors, livestock pathogen reservoirs, and the risk of human giardiasis. Our findings of a late summer peak in incidence are in agreement with previous studies examining temporal trends in human giardiasis (Addiss et al. 1992; Naumova et al. 2007; Keegan et al. 2009; Lal et al. 2012). The consistency of a seasonal pattern, along with the persistence of *Giardia* as a waterborne pathogen (Yaoyu and Xiao 2011), suggests human giardiasis is sensitive to climate and hydrology factors.

Associations between human case occurrence and hydrological variables were identified in both the multivariable and case crossover analysis. Inverse associations between aggregated human case counts and river water levels have been previously reported for another waterborne pathogen, *Legionella*, in multivariable models (Ng et al. 2008). In contrast to the findings of our multivariable model, the case crossover analysis found that the odds of human cases increased with the water level and flow of the Grand River occurring 1 week before cases. These findings differ from a case crossover study on the enteric protozoa, *Cryptosporidium*, which identified increased water flow and water levels as having a protective effect on human case risk and attributed this to dilution of pathogen concentrations in the watershed (Brankston et al. 2018). The discrepancy between these results may be related to external control of river flow and level exercised by the Grand River Conservation Authority via a system of dams and water reservoirs which minimize flooding risk and maintain water quality for discharging sewage plants. The Grand River at Doon, Ontario, where hydrology data for this study was collected, is heavily augmented during the late summer dry period. Up to 90% of the total river water volume at Doon in August can come from the reservoir (Grand River Conservation Authority 2014). While the effects of river level manipulation may be lost in monthly averaged exposure data, reservoir release seen as an acute exposure could influence associations being found with human case counts using the case crossover methodology. Alternately, resuspension of enteric pathogens from stream sediments during periods of high water flow, such as stormy weather, has been proposed as an explanation for measurements of increased concentrations of *E. coli* occurring with high stream flow rates (Dorner et al. 2006). While an explanatory hypothesis for a positive association between human giardiasis cases and water levels and flow rates in relation to increased river cyst concentration is unclear in the present study, cyst survival in river sediments and potential for resuspension is an area for future research. When the presence of livestock reservoirs was added to the model (Objective 2), an increase in the odds of human cases was seen with a 1-week lag period. Surveillance studies have confirmed the presence of zoonotic *Giardia* assemblages in Ontario cattle and swine herds (Coklin et al. 2007; Dixon et al. 2011; Farzan et al. 2011), providing initial support for the hypothesis of spill-over of zoonotic *Gd* from livestock reservoirs. Our results would benefit from molecular typing of *Giardia* cysts obtained from the Grand River to confirm if potentially zoonotic cysts from livestock can survive effluent transport mechanisms into the watershed.

Statistically significant associations between mean temperature and human case incidence from the case crossover analysis resulted in inverse effects at the lowest tertile of mean temperature with a lag period of 3 weeks. In both the full and truncated time series, the lowest mean air temperatures occurring three weeks prior to human cases decreased the risk of giardiasis.

Previous studies have reported associations between time-lagged high temperatures and increased risk of waterborne enteric disease (Thomas et al. 2006; Naumova et al. 2007; Brankston et al. 2018), possibly reflecting changes in human behaviour and greater opportunities for exposure to contaminated surface waters (Thomas et al. 2006; Peng et al. 2008; Brankston et al. 2018). While the results found in this study do not directly support this hypothesis, it is possible that on days with cooler than average temperatures a protective effect is observed as people may be less inclined to recreate in natural water bodies.

In the truncated time series, within-stratum highest precipitation levels occurring 4 weeks prior to human cases were inversely related to giardiasis occurrence (Table 2). This result contradicts two studies that found that high rainfall events preceded increased human enteric case incidence (Thomas et al. 2006; Escobedo et al. 2015), however, one study was based in Cuba where average rainfall is higher than what is observed in Ontario (Escobedo et al. 2015). The second study, based in Canada, used a longer hazard period of 6 weeks and stratified precipitation to assess only extreme rainfall events (Thomas et al. 2006). The difference in results may be related to stratification choice, length of hazard period, or pathogen biology.

Some limitations should be noted. Aggregating cases into monthly counts could mask underlying patterns. For example, environmental variables averaged by month may not reflect the true exposures influencing transmission, potentially leading to imprecise inferences. Case crossover analysis aims to avoid this potential bias by examining associations between acute exposures and daily case occurrence using a self-matched study design. However, the testing of multiple hypotheses in the case crossover method may increase the probability of a Type 1 error resulting in spurious associations. In this study, type 1 errors were minimized by grouping exposures to minimize the number of hypotheses tested.

The livestock sampling data for *Gd* was constrained by inconsistent monthly sampling and sporadic genotyping results, leading to assemblage type reporting for just over 50% of positive samples. We acknowledge that the *Giardia*-positive farm counts might not accurately reflect the true number of *Giardia*-positive farms in the region. Nonetheless, the positive counts taken from surveillance data represent a subset and likely underrepresent the true number of cases, influencing the bias towards the null. Assemblage typing data provided confirmation that potentially zoonotic *Gd* assemblages were present in livestock reservoirs in the region, supporting the possibility of a zoonotic transmission route. Molecular studies of isolates and genetic assemblage typing of samples would strengthen the spill-over hypothesis. Effect modification between positive-testing farms and environmental variables was not investigated due to limited farm data, however including this as a future direction could contribute to our understanding of livestock disease reservoirs.

Symptom onset dates were reported for 75% (Objective I dataset) and 73% (Objective II dataset) of human case counts used in this study. The remaining cases were included in the study to counteract underreporting which is known to occur however, it is possible that the inclusion of these cases resulted in incorrect estimates of associations and exposure lag periods.

Ecological systems are changing at an unprecedented rate (Walther et al. 2002; Post et al. 2009). With climatic conditions expected to change in the coming years, it is necessary to understand the environmental drivers of climate-sensitive diseases (Bouley et al. 2014; Machalaba et al. 2015). While the design of this study precludes conclusions being drawn regarding cause and effect, the relationships described provide an understanding of the associations between human giardiasis, livestock disease reservoirs, and environmental variables using a one health approach.

## Conclusion

Seasonality of human *Gd* cases appears to be influenced by hydrology and temperature variables occurring 1–3 weeks prior to case occurrence. Molecular epidemiology confirming survival of zoonotic pathogen spill-over from livestock into watersheds would assist in clarifying the influence of livestock reservoirs. The results of this study contribute information about the influence of environmental and livestock exposures on a climate-sensitive waterborne disease and may be useful in future forecasting of the effects of climate change on human enteric disease.

## Electronic supplementary material

Below is the link to the electronic supplementary material.

Supplement 1 Age distribution of confirmed *Giardia duodenalis* cases in people reported to the Integrated Public Health Information System for Waterloo Health Region, Ontario, 2006 – 2013. (PNG 33 kb)

Supplement 2 Daily weather and hydrological observations from Waterloo Health Region, Ontario, Canada, 1 June 2006 – 31 December 2013. A: Mean Air Temperature (°C); B: Total Precipitation (mm); C: Water Flow Rate of the Grand River (m^3^/s); D: Water Level of the Grand River (m). (JPEG 114 kb)

Supplement 3 Complete case crossover results evaluating associations between environmental exposures and human cases of *Giardia duodenalis* in the Waterloo health region, Ontario, 2006 – 2013 (Objective 1). (JPEG 269 kb)

Supplement 4 Complete case crossover results evaluating associations between environmental exposures, livestock reservoirs and human cases of *Giardia duodenalis* in the Waterloo health region, Ontario, 2006 – 2008 (Objective 2). (JPEG 311 kb)
