# Supplementary figures and images for: The Influence of Climate and Livestock Reservoirs on Human Cases of Giardiasis
Source: Ecohealth. 2018 Oct 22;16(1):116–27. doi: 10.1007/s10393-018-1385-7 (PMC6430827; doi:10.1007/s10393-018-1385-7)

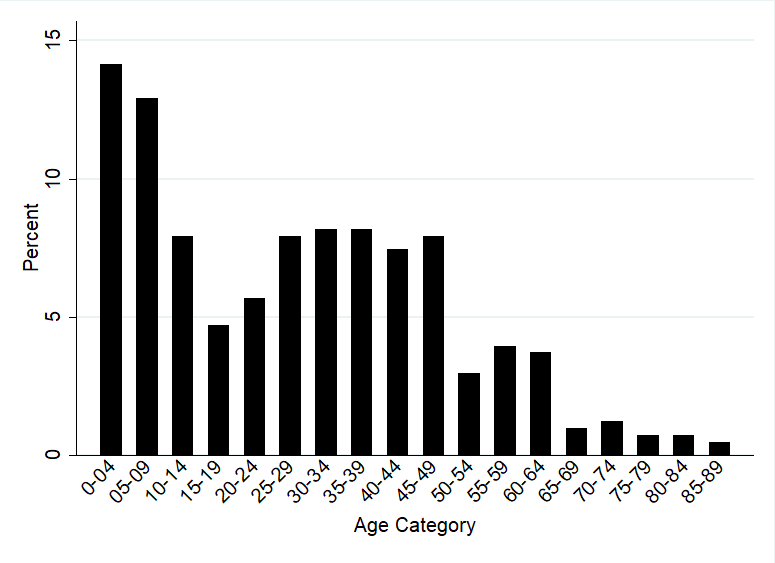

Supplement: Supplementary file 1 — Age distribution of confirmed Giardia duodenalis cases in people reported to the Integrated Public Health Information System for Waterloo Health Region, Ontario, 2006 – 2013. (PNG 33 kb) [file 10393_2018_1385_MOESM1_ESM.png]

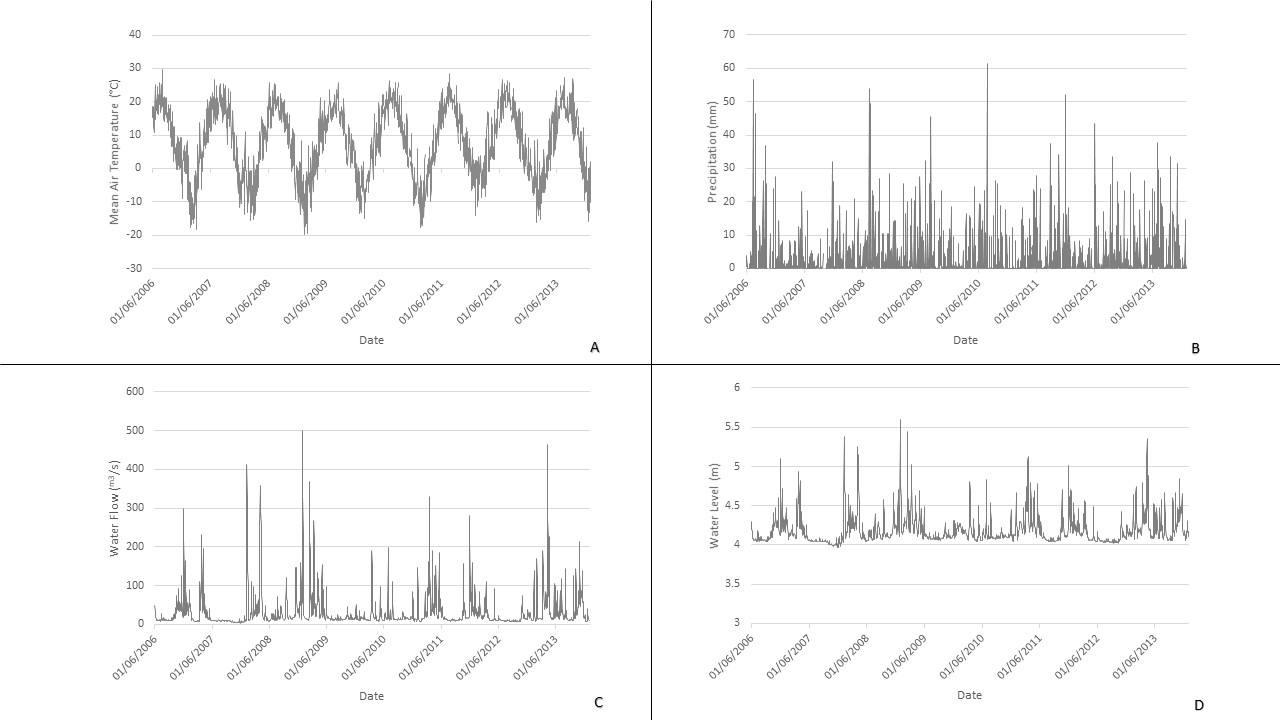

Supplement: Supplementary file 2 — Daily weather and hydrological observations from Waterloo Health Region, Ontario, Canada, 1 June 2006 – 31 December 2013. A: Mean Air Temperature (°C); B: Total Precipitation (mm); C: Water Flow Rate of the Grand River (m3/s); D: Water Level of the Grand River (m). (JPEG 114 kb) [file 10393_2018_1385_MOESM2_ESM.jpg]

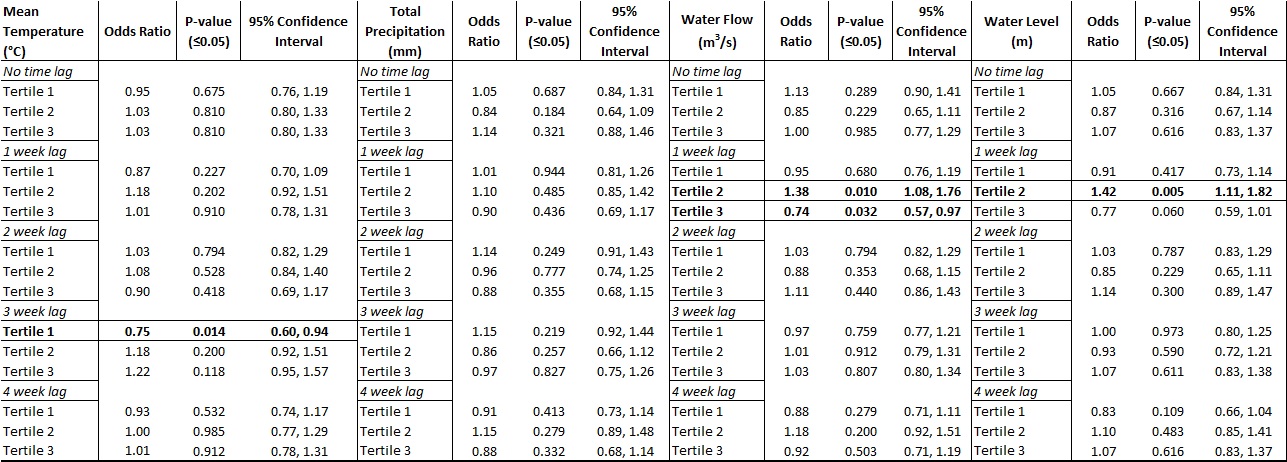

Supplement: Supplementary file 3 — Complete case crossover results evaluating associations between environmental exposures and human cases of Giardia duodenalis in the Waterloo health region, Ontario, 2006 – 2013 (Objective 1). (JPEG 269 kb) [file 10393_2018_1385_MOESM3_ESM.jpg]

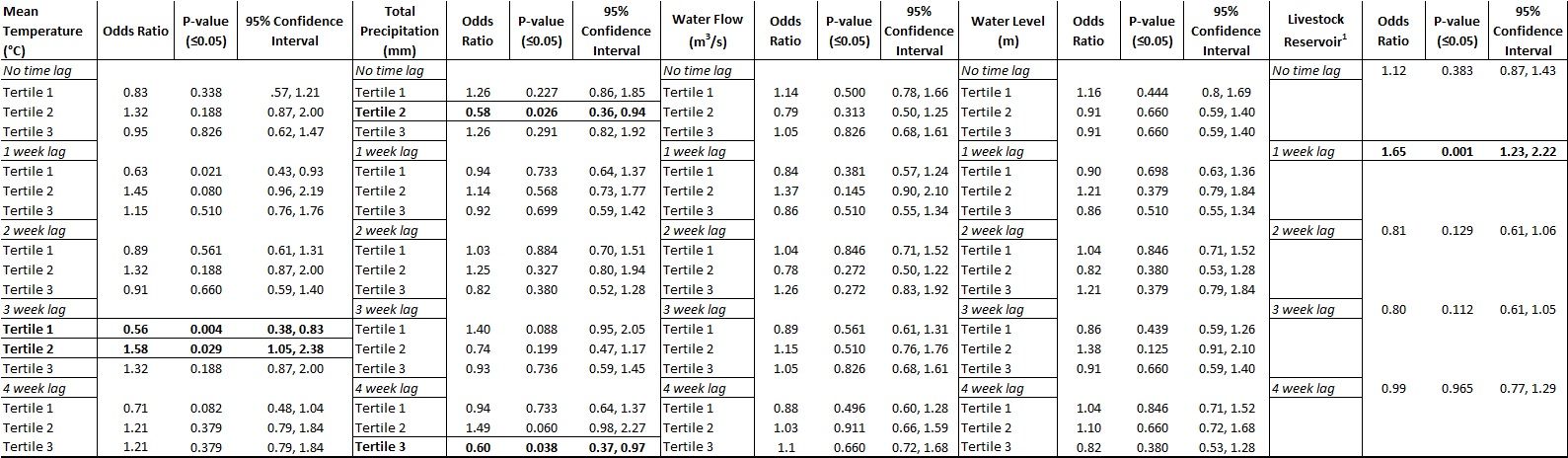

Supplement: Supplementary file 4 — Complete case crossover results evaluating associations between environmental exposures, livestock reservoirs and human cases of Giardia duodenalis in the Waterloo health region, Ontario, 2006 – 2008 (Objective 2). (JPEG 311 kb) [file 10393_2018_1385_MOESM4_ESM.jpg]
